# 3D dynamic cultures of HGSOC organoids to model innovative and standard therapies

**DOI:** 10.3389/fbioe.2023.1135374

**Published:** 2023-04-18

**Authors:** Enrico Cavarzerani, Isabella Caligiuri, Michele Bartoletti, Vincenzo Canzonieri, Flavio Rizzolio

**Affiliations:** ^1^ Pathology Unit, Centro di Riferimento Oncologico di Aviano (C.R.O.) IRCCS, Aviano, Italy; ^2^ Department of Molecular Sciences and Nanosystems, Ca’ Foscari University of Venice, Venice, Italy; ^3^ Unit of Medical Oncology and Cancer Prevention, Department of Medical Oncology, Centro di Riferimento Oncologico di Aviano (CRO), IRCCS, Aviano, Italy; ^4^ Department of Medical, Surgical and Health Sciences, University of Trieste, Trieste, Italy

**Keywords:** HGSOC, microfluidic technology, cancer organoids, therapy, Pin1

## Abstract

High-grade serous ovarian cancer (HGSOC) needs new technologies for improving cancer diagnosis and therapy. It is a fatal disease with few options for the patients. In this context, dynamic culture systems coupling with patient-derived cancer 3D microstructures could offer a new opportunity for exploring novel therapeutic approaches. In this study, we optimized a passive microfluidic platform with 3D cancer organoids, which allows a standardized approach among different patients, a minimum requirement of samples, multiple interrogations of biological events, and a rapid response. The passive flow was optimized to improve the growth of cancer organoids, avoiding the disruption of the extracellular matrix (ECM). Under optimized conditions of the OrganoFlow (tilting angle of 15° and an interval of rocking every 8 min), the cancer organoids grow faster than when they are in static conditions and the number of dead cells is reduced over time. To calculate the *IC*_*50*_ values of standard chemotherapeutic drugs (carboplatin, paclitaxel, and doxorubicin) and targeted drugs (ATRA), different approaches were utilized. Resazurin staining, ATP-based assay, and DAPI/PI colocalization assays were compared, and the *IC*_*50*_ values were calculated. The results showed that in the passive flow, the *IC*_*50*_ values are lower than in static conditions. FITC-labeled paclitaxel shows a better penetration of ECM under passive flow than in static conditions, and cancer organoids start to die after 48 h instead of 96 h, respectively. Cancer organoids are the last frontiers for *ex vivo* testing of drugs that replicate the response of patients in the clinic. For this study, organoids derived from ascites or tissues of patients with Ovarian Cancer have been used. In conclusion, it was possible to develop a protocol for organoid cultures in a passive microfluidic platform with a higher growth rate, faster drug response, and better penetration of drugs into ECM, maintaining the samples’ vitals and collecting the data on the same plate for up to 16 drugs.

## Introduction

Deadly diseases are characterized by a complicated molecular network of players that are difficult to treat (Hanahan and Weinberg, 2011). High-grade serous ovarian cancer (HGSOC) belongs to this category of pathologies and no resolutive therapeutic approaches are available (Bowtell et al., 2015). The 5-year survival rate is less than 30% for metastatic diseases with a median overall survival rate of approximately 11 months (Moore et al., 2018). Both primary debulking surgery or neoadjuvant chemotherapy followed by interval debulking surgery are the main approaches, coupled with the mutational status of homologous recombination-deficiency and poly-ADP ribose polymerase (PARP) inhibitors (Moore et al., 2018).

Several innovative therapeutic approaches are currently underway, including combined immunotherapies with PARP inhibitors, low-dose radiotherapy, epigenetic and anti-angiogenic drugs, or inflammation restoration (e.g., by reprogramming stromal cells), but are still not curative (Kandalaft et al., 2022). The reasons are different and include the unknown origin of the disease and no appropriate 2D cell cultures and animal models, at least until recent years. Indeed, large tumors have been found on and around the ovaries suggesting that its surface is the origin of the pathology, as first proposed by the British surgeon Thomas Spencer Wells in 1873 (Wells, 1873). But in the mid-2000s, starting from the observations of the pathologist Christopher Crum, many studies identified serous tubal intraepithelial carcinoma (STIC) (Piek et al., 2001) in the fallopian tubes of people with ovarian cancer risk genes, especially at the nearest end of the ovaries (Lee et al., 2007; Norquist et al., 2010). Although STIC is observed in no more than 60% of people with HGSOC (Przybycin et al., 2010), the fallopian tubes are the main tissues of origin. The transformed fallopian tube secretory epithelial cells, when injected into the peritoneum of nude mice, induce tumors that resemble human HGSCs (Karst et al., 2011) (Jazaeri et al., 2011). Mouse model studies confirmed this evidence, and by genetic manipulations of the fallopian tube, epithelial cells could be progressively transformed into HGSOC cells (Perets et al., 2013). Previous genetic models generated from the manipulation of the ovarian surface epithelium did not represent the overall HGSOC characteristics (Kinross et al., 2012; Szabova et al., 2012).

Even the most utilized cell lines in the 2D culture were not fully appropriate to replicate the complexity of HGSOC (Domcke et al., 2013). In an in-depth comparison of 47 cell lines of copy-number changes, mutations and mRNA expression profiles revealed that underutilized cell lines (e.g., Kuramochi and Ovasaho) closely recapitulate HGSOC more than in the statistically overrepresented A2780 and Skov-3 cell lines (Domcke et al., 2013).

Even though more appropriate *in vivo* and *ex vivo* models are in place (Ciucci et al., 2022) and next-generation technology is revealing the most important genetic and genomic alterations that could be druggable (Cheng et al., 2022), it remained challenging to improve human *ex vivo* models that could facilitate bench-to-bed drug testing and dictate the right drug for every single patient in a short time frame. Also, nanotechnology that is revolutionizing medicine (Decuzzi et al., 2021) still suffers from not perfect models for intelligent nanodrug testing (Bhatia et al., 2022). The lack of suitable models to study OC prompted scientists to develop primary patient-derived xenografts (PDX). This model is extremely valid in replicating the histopathological characteristics of the patients (Weroha et al., 2014) and is able to replicate the patient’s response to chemotherapy (Glaser et al., 2015). However, it is extremely expensive, time-consuming, and difficult to use, and it raises some ethical considerations.

A step forward was the development of organoid technology, 3D cultures of stem cells-derived differentiated cells that recapitulate the original organ (Sato et al., 2009) or tumor (cancer organoids) (Van De Wetering et al., 2015), that was utilized in basic research (Scattolin et al., 2020a; Scattolin et al., 2020b; Granchi et al., 2021; Tzouras et al., 2022) and even in clinical trials (Vivarelli et al., 2020). Specifically, on ovarian cancer organoids, two seminal papers reported that this technology could be used for the functional profiling of DNA repair, predicting responses to PARP inhibitors (Hill et al., 2018), and providing a long-term expansion of organoids. The technology could also be used in representing all main subtypes of ovarian cancer, capturing different tumor subtype responses to gold-standard platinum-based chemotherapy, and achieving chemoresistance in recurrent diseases (Kopper et al., 2019). But in most of the published studies, cancer organoids are utilized to screen current and novel drugs in static conditions.

Microfluidic technology combined with organoids has the power to close the gap in the complexity of the human body (Duzagac et al., 2021; Saorin et al., 2022). In a microfluidic organoid-on-a-chip platform, flow conditions, nutrient supply, shear stress, input-output, and geometry can be easily controlled, providing a way to carry out the experiment from a cellular to tissue and, finally, to organ levels (Duzagac et al., 2021). A critical step in the drug development and testing process is to apply sufficiently accurate models that capture key aspects of the human body and tumor physiology. Conventional models fail to recapitulate the complexity of organ physiology. Innovative studies demonstrate the power of microfluidics to develop robust microfluidic organoid-on-a-chip models for investigating the tumor and its response to drugs. Some research groups were able to develop microfluidics 3D advanced cell cultures, including inflammatory bowel disease (Beaurivage et al., 2019), an *in vitro* 3D blood-brain barrier model system equipped with brain microvascular endothelial cells derived from human induced pluripotent stem cells used for drug development (Kurosawa et al., 2022), an innovative model to predict responses to therapy in breast cancer (Lanz et al., 2017), and hepatocytes in the Mimetas OrganoPlate (Jang et al., 2015). However, HGSOC organoids derived from patients combined with microfluidics technology were not investigated.

However, the advantages that microfluidics could provide to 3D systems in creating more representative models of human cancers, such as lung (Stucki et al., 2015), liver (Gori et al., 2016), intestine (Pocock et al., 2017), and heart (Agarwal et al., 2013) is even applicable in HGSOC. In particular, the possibility to control extremely small volumes (in the order of the microliters) with extreme precision allows microfluidic technology to create *in vitro* models that are considerably closer to the conditions that exist in the human body. Since it is possible to control mechanical stimuli such as shear and stretch forces, it is feasible to create vascular models by generating flows in these vessels (Wang et al., 2016; Lee et al., 2018). The traditional system of cultivating organoids (static cultures) limits the size of the organoids by reducing the growth rate. In fact, necrotic phenomena are common in the inner core of the organoids due to an insufficient supply of oxygen, nutrients, and essential metabolites. These problems can be overcome by the use of a microfluidic system, which, by mimicking the vasculature, allows long-term cultures of organoids to be made (Yamada et al., 2012). Research has found that the transportation of nutrients, essential metabolites, and oxygen by laminar flow reduces the size of the necrotic core, favoring the increase in the size of the organoids and improving the organ functionality (Berger et al., 2018; Homan et al., 2019).

In this paper, we combined a passive microfluidic technology and cancer organoids derived from HGSOC patients in a dynamic culture with a targeted drug to test chemotherapeutic drugs and a nanodrug utilized in clinics with the idea to develop a rapid, physiological, and non-destructive tool for a patient-tailored approach.

## Materials and methods

### Cancer organoids culture

Organoids were obtained from totally anonymized specimens. However, biobank informed consent for research purposes was obtained to collect the samples at the National Cancer Institute (CRO) of Aviano.

The ascitic fluids were centrifuged at 1,000 rpm for 10 min and the pellets of cells were washed twice with HBSS (Gibco, Massachusetts, United States). In order to lyse erythrocytes present in the fluid, cold red blood cell lysis buffer (Roche Diagnostics, Basel, Switzerland) was added and kept on ice under stirring for 10 min. The pellet was centrifuged at 1,000 rpm for 10 min and resuspended in Cultrex RGF BME, Type 2 (Bio-techne, Minnesota, USA). Solid tumor tissues were incubated in Dulbecco’s modified Eagle’s medium/Nutrient Mixture F-12 Ham implemented with Levofloxacin 100 μg/mL, Vancotex 25 μg/mL, Ciproxin 5 μg/mL, Gentamicin 200 μg/mL, and Fungizone 5 μg/mL for 30 min. The tissues were minced into approximately 0.5–1 mm diameter pieces with fine dissection scissors, and 1 mL of 4 mg/mL collagenase IV (Gibco, Massachusetts, United States) solution was added, incubated at 37°C for no more than 45 min, and mechanically dissociated by pipetting. The clusters of cells were centrifuged at 1,000 rpm for 10 min, resuspended in an appropriate volume of Cultrex RGF BME, Type 2 (Bio-techne, Minnesota, United States) and seeded on a 24-well cell culture plate. After the polymerization of Cultrex, 500 μL of organoid culture medium, as described by Kopper et al., was added to each well and refreshed every 3 days (Kopper et al., 2019). The organoids were cultured at 37°C and 5% of CO_2_.

### Immunohistochemistry

Sections of formalin-fixed, paraffin-embedded ascites and solid tumor patient-derived organoids were used for histopathological analyses. Organoids were recovered from BME using ice-cold cell recovery solution (Corning, New York, United States) in accordance with manufacturing protocols, fixed in phosphate-buffered 10% formalin, and embedded in 500 μL of Bio-Agar (Bio-Optica Milano Spa, Milano, ITA). Five μm sections were stained with hematoxylin and eosin (H&E) using a Leica ST5020 multistainer and 2 μm sections were cut for IHC analysis. The IHC was performed with an UltraVision LP Detection System HRP DAB kit (Thermo Scientific, Waltham, USA). Heat-induced antigen retrieval was performed using 10 mM citrate buffer pH 6.0. The following antibodies were used to characterize HGOC patients-derived organoids and parental tumors: PAX8 (ProteinTech Group, Germany, EU), WT1 (Abcam, U.K.), and CA-125 (Santacruz Biotechnology, TX).

### Immunofluorescence

The organoids were characterized by immunofluorescence following the protocol of Dekkers et al. (Dekkers et al., 2019). The following primary antibodies were used to characterize HGOC patients-derived organoids culture in static and passive flow conditions: PAX8 (ProteinTech Group, Germany, EU), WT1 (Abcam, U.K.), and CA-125 (Santacruz Biotechnology, TX). The secondary antibodies that were used are Goat anti-Rabbit IgG (H + L) Alexa Fluor™ Plus 488 (Thermo Fisher Scientific Waltham, Massachusetts, United States) and Goat anti-Mouse IgG (H + L) Alexa Fluor™ Plus 488 (Thermo Fisher Scientific Waltham, Massachusetts, United States). The images were acquired with an EVOS FL Auto 2 (Thermo Fisher Scientific Waltham, Massachusetts, United States).

### Experimental conditions for organoids cultures

**FIGURE 1:**
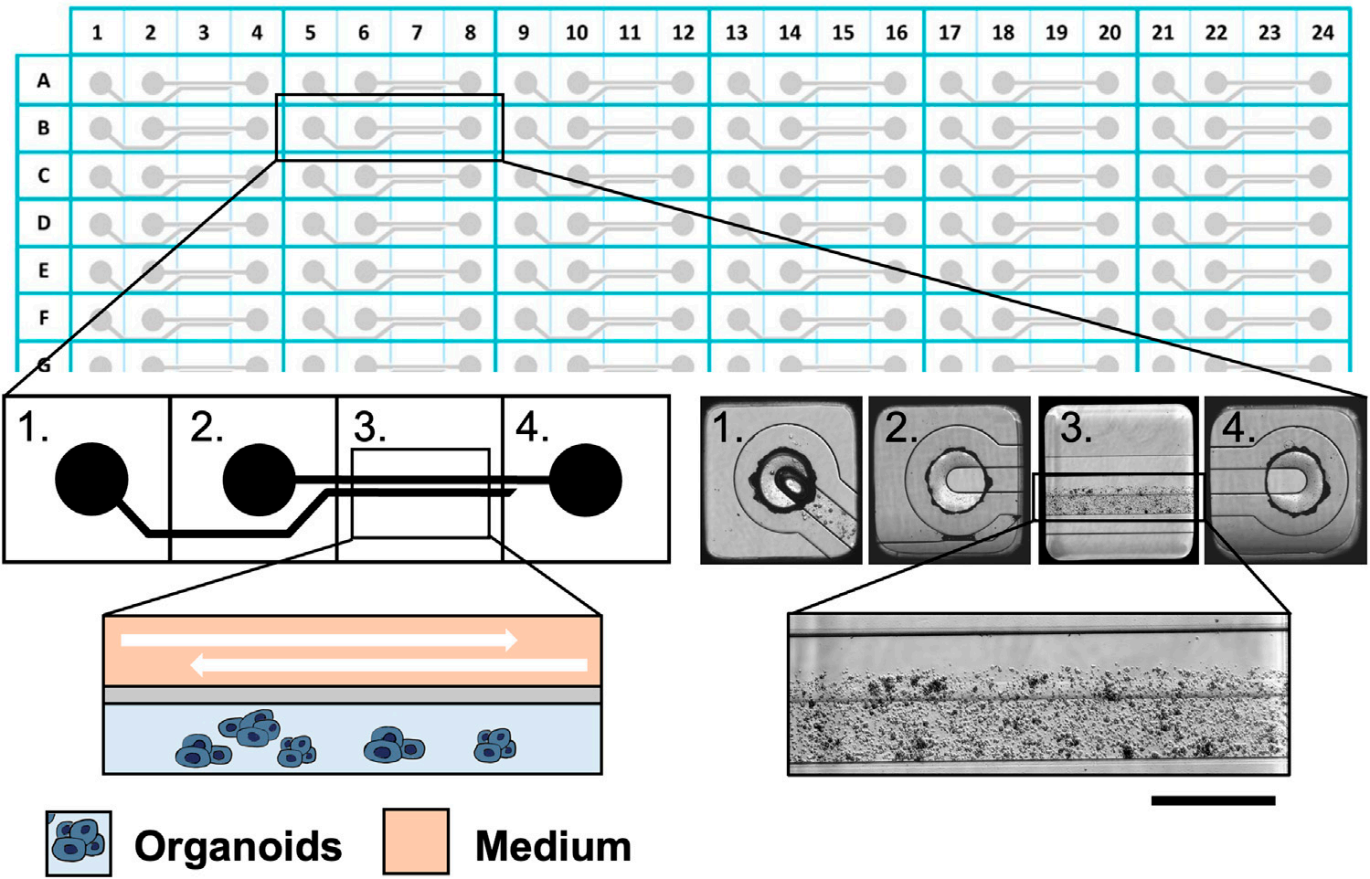
The Mimetas 2-lane OrganoPlate^®^ is a 384-well plate composed of 96 independent tissue culture chips. A graphical representation of the chip is provided on the left, while bright field images are shown on the right. Each chip is composed of 4 units: 1) the gel inlet, 2) the medium inlet 3) the observation window, and 4) the medium outlet. In the observation window, it is possible to distinguish two microfluidic channels: the upper one in which the medium flows and the lower one which are the organoids in the ECM. Scalebar is 250 μm.”

The Mimetas 2-lane OrganoPlate^®^ consists of 384 well plates composed of 96 independent tissue culture chips. The medium perfusion is provided by gravity through the microfluidic channels of the OrganoFlow^®^, a pump- and tube-free perfusion basculating platform with an adjustable time and tilting angle and interval rocking settings. In order to establish better conditions for cultured organoids in the Mimetas 2-lane OrganoPlate^®^, a comparison with the static culture in the 96-wells plates was done. Clusters of organoids were mixed in an appropriate volume (approximately 10,000 clusters of cells/mL) of Cultrex RGF BME, Type 2 (Bio-techne, Minnesota, United States) previously diluted 1:1 with DMEM/F12 without phenol red (SIGMA-ALDRICH, Missouri, United States), and 2 uL of this mixture in five replicates was seeded in 96-wells plates and every chip of the Mimetas OrganoPlate^®^ 2-lane 96. In particular, the mixture of organoids, Cultrex, and DMEM/F12 was loaded in the “Gel Inlet” channel of the OrganoPlate^®^ 2-lane 96, and after the polymerization of the mixture, 50 μL of the organoid medium was loaded in the “Medium inlet” channel and 50 μL in the “Medium outlet” channel (Figure 1). Flow and inclination rates were evaluated and compared with the static cell cultures in the 96-wells plates. There were three different tilting angles of 7°, 15°, and 20° each with four different intervals of rocking every 30 s; 1, 8, 40 min, and continuous rocking were compared. The viability of organoids in dynamic and static conditions was evaluated at 0, 24, 72, and 96 h with the PrestoBlue HS Assay (Invitrogen Corporation, San Diego, United States) in accordance with manufacturing protocols. The viability was performed in seven replicates and the *p*-value was calculated using two-way ANOVA with GraphPad Prism (La Jolla, CA, United States). The *p*-values are expressed as follows: * *p* ≤ 0.05, ** *p* ≤ 0.01, *** *p* ≤ 0.001, and **** *p* ≤ 0.0001.

### Organoid viability

In order to assess the viability of the organoids to evaluate differences in proliferation and necrosis between the static and the dynamic cultures, the LIVE/DEAD^®^ Viability/Cytotoxicity Kit (Thermo Fisher Scientific Waltham, Massachusetts, United States) was used. The viability of the organoids was evaluated with or without 50 μM Carboplatin at 72 and 96 h. In particular, the organoids were stained for 40 min with Calcein AM 0,50 μM, washed twice with HBSS, and stained with EthD-1 0,25 μM for 40 min. After being washed twice with HBSS, 100 μL DMEM/F12 without phenol red was applied and the fluorescence was observed in a Nikon Eclipse Ts2R. The green (live organoids cells) and red (dead organoids cells) pixels were calculated and were normalized on the blue pixels (total number of cells) with ImageJ.

To evaluate the penetration of the drug into the ECM in static and dynamic conditions, organoids were treated with 1 μM Oregon Green™ 488 Paclitaxel (Thermo Fisher Scientific Waltham, Massachusetts, United States). The Fluo-Paclitaxel was monitored over time at 3, 24, 48, 72, and 96 h. The organoids were stained with Hoechst 33,342 (Thermo Fisher Scientific Waltham, Massachusetts, United States) for 20 min, washed twice with HBSS, and stained with 5 μg/mL of Propidium iodide (Thermo Fisher Scientific Waltham, Massachusetts, United States) for 15 min. After being washed twice with HBSS, 100 μL DMEM/F12 without phenol red was applied and the fluorescence was observed in a Nikon Eclipse Ts2R.

The experiments were performed in triplicates and the *p*-value was calculated using a two-tailed Student’s *t*-test with GraphPad Prism (La Jolla, CA, United States). The *p*-values are expressed as follows: * *p* ≤ 0.05, ** *p* ≤ 0.01, *** *p* ≤ 0.001, and **** *p* ≤ 0.0001.

### Paclitaxel quantification

A triplicate of 2 μL of 1:1 Cultrex/DMEM-F12 was seeded in a 96-wells plate and in the Mimetas OrganoPlate^®^ 2-lane 96. After the polymerization, a solution of 10 μM Oregon Green™ 488 Paclitaxel in DMEM/F12 was added. Every 24 h, the solution was completely removed and the fluorescence of the Oregon Green™ 488 Paclitaxel that remained in the supernatant was measured with a BioTek Synergy H1. The *p*-value was calculated using a two-tailed Student’s *t*-test with GraphPad Prism (La Jolla, CA, US), and the *p*-values are expressed as follows: * *p* ≤ 0.05, ** *p* ≤ 0.01, *** *p* ≤ 0.001, and **** *p* ≤ 0.0001.

### Half-maximal inhibitory concentration (IC_50_)

Clusters of organoids were mixed in an appropriate volume of Cultrex RGF BME, Type 2 previously diluted 1:1 with DMEM/F12 without phenol red, and 2 μL of this mixture was seeded in 96-wells plates and every chip of the Mimetas OrganoPlate^®^ 2-lane 96. The viability was measured with the PrestoBlue HS Assay every 24 h for 1 week (168 h) in accordance with manufacturing protocols. The viability was performed in four replicates and the *p*-value was calculated using a two-tailed Student’s t-test with GraphPad Prism (La Jolla, CA, United States). The *p*-values are expressed as follows: * *p* ≤ 0.05, ** *p* ≤ 0.01, *** *p* ≤ 0.001, and **** *p* ≤ 0.0001.

After the viability assays were performed, the organoids were treated with six different concentrations of Carboplatin, ATRA (all-trans Retinoic acid), Paclitaxel (200, 40, 8, 1.6, 0.32, and 0.064 μM), Doxorubicin (50, 10, 2, 0.4, 0.08, and 0.016 μM), and Caelyx (100, 20, 4, 0.8, 0.16, and 0,032 μM). After 96 h, cell viability was measured using three different methods. ATP-based assay: the CellTiter-Glo 3D (Promega, Madison, WI, United States) was used to calculate the viability, and the luminescence was acquired with BioTek Synergy H1. Reducing-based assay: the PrestoBlue HS assay was used to calculate the viability, and the fluorescence was acquired with BioTek Synergy H1. Florescence imaging-based assay: the organoids were stained with 100 μL of 0,1 μg/mL of Hoechst 33,342 (Thermo Fisher Scientific Waltham, Massachusetts, United States) in DMEM/F12 without phenol red and incubated for 20 min. After the incubation, the organoids were washed twice with HBSS and stained with 100 μL of 5 μg/mL of Propidium Iodide (Thermo Fisher Scientific Waltham, Massachusetts, USA) in DMEM/F12 without phenol red and kept for 15 min. The medium was removed, and the organoids were washed twice with HBSS, so 100 μL of DMEM/F12 without phenol red was applied and the fluorescence was observed in a Nikon Eclipse Ts2R. The colocalization of blue areas (nuclei of organoids) and red areas (dead cells of organoids) were evaluated with Pearson’s correlation coefficient using the ImageJ software.

For all three methods, luminescence, fluorescence, and Pearson’s correlation coefficient outcome were used to establish the logistical dose-response curves and calculate the IC_50_ with GraphPad Prism (La Jolla, CA, US).

## Results and discussion

### Microfluidic setup for the optimal growth of HGSOC organoids

**FIGURE 2:**
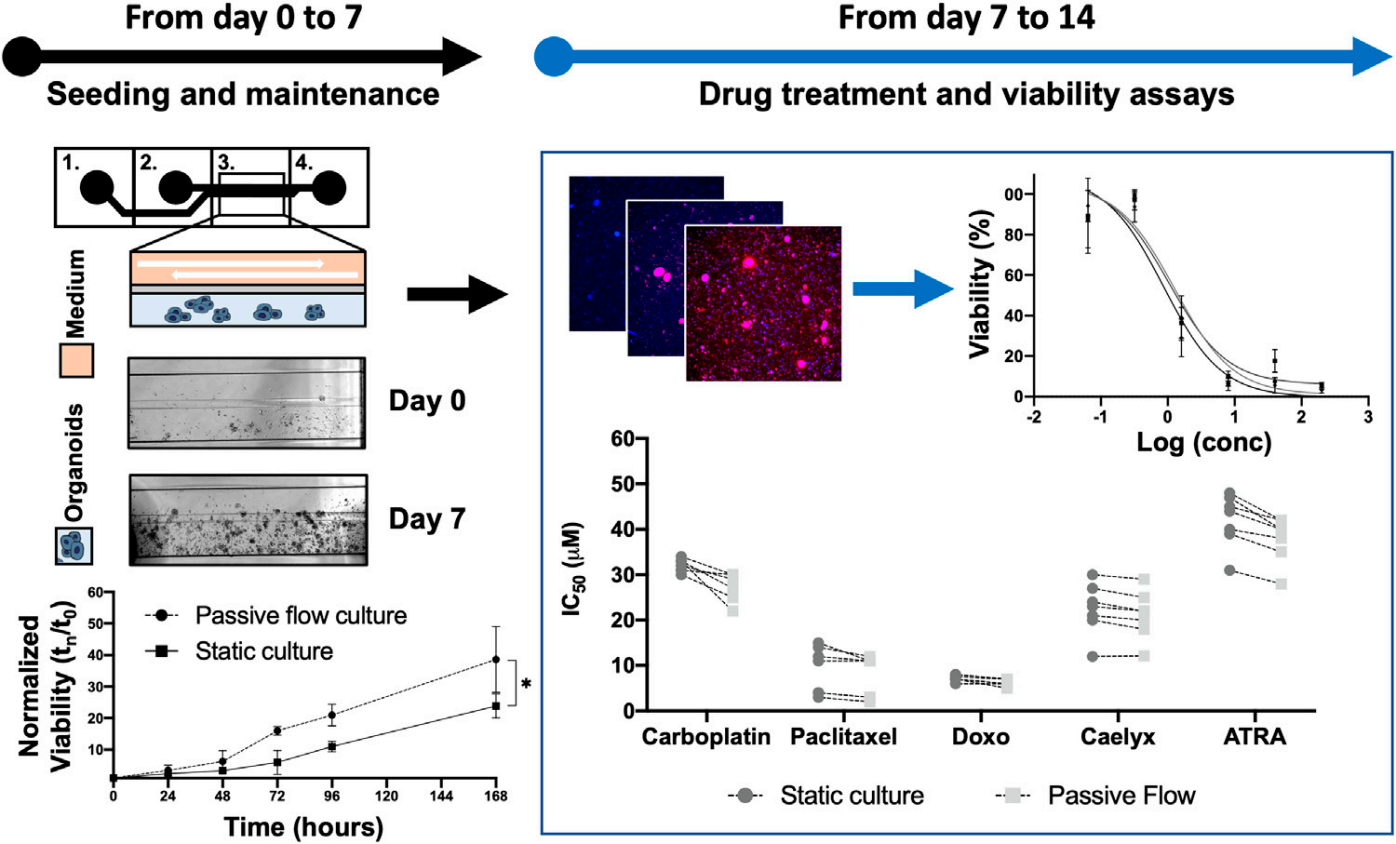
Flowchart: Previously, a protocol to cultivate the organoids in the Mimetas OrganoPlate^®^ 2-lane 96 was defined. Organoids were seeded both in Mimetas and in 96-wells plates (day 0) and were maintained in culture for 7 days (day 7). The viability was measured every day for 7 days and the growth curve was established. After 1 week (day 7), the organoids were incubated with the drugs for 96 h. At the end of the incubation time, cell viability was measured (with ATP-based, Resazurin-based, and Fluorescence-based assays); the dose-response curve was established and IC_50_ values were calculated (both in dynamic and static cultures). The *p*-value was calculated using two-way ANOVA, * *p* ≤ 0.05.

As the first step, the experiments were set up to demonstrate the superiority of dynamic versus static cultures with organoids directly derived from HGSOC patients for the first time. Figure 2 shows a flowchart of the designed experiments with the intended goals.

**FIGURE 3:**
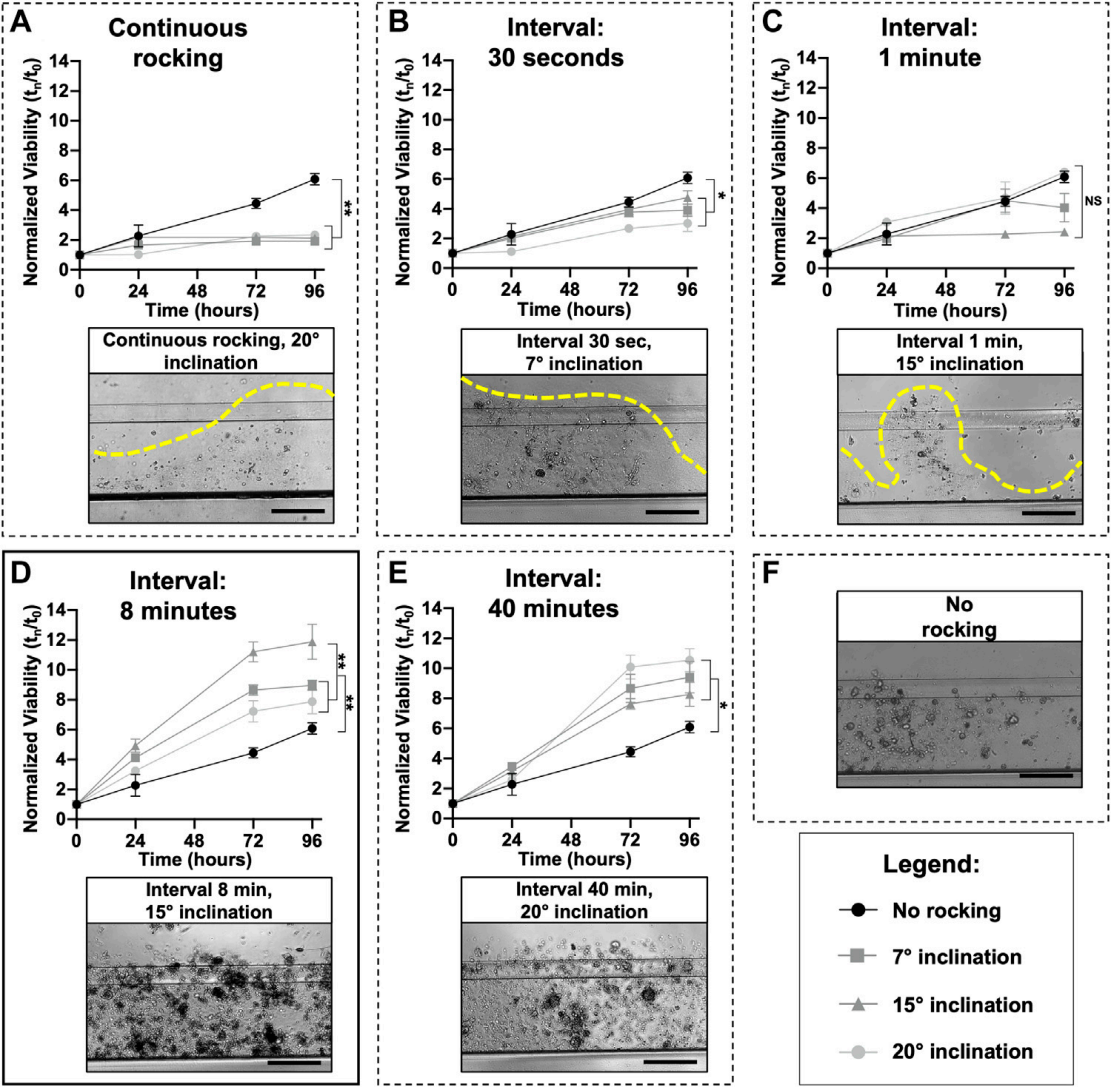
Microfluidic setup: The perfusion flow of the medium inside the OrganoFlow can be modulated by adjusting the interval of rocking (switching sides every 5 s to 999 min) and rocking angles (0° to +25°); to find the best growth conditions, different microfluidic setups were tested. For each rocking condition, the growth curves of organoids divided by three different tilting angles (7°, 15°, and 20°) and compared with the no rocking condition (in black) is reported; the same was done for all the conditions. Cell viability (mean ± SD) was measured using the PrestoBlue HS Assay for 96 h. A representative bright-field image of organoids cultured in different conditions is also shown. In particular, image **(D)** corresponds to the condition (tilting angle of 15° and interval of rocking every 8 min) in which the organoids have the highest growth rate, while **(E)** corresponds to the interval of rocking of 40 min. Images **(A–C)** correspond to continuous rocking and intervals of rocking of 30 s and 1 min, respectively; all these conditions generate a very high flow that destroys the ECM. Image **(F)** corresponds to a no-rocking condition. The *p*-values were calculated using two-way ANOVA, * *p* ≤ 0.05, ** *p* ≤ 0.01. Scale bar 500 μm.

Different research laboratories have shown that microfluidic technology could outperform static conditions due to a better exchange of media, elimination of waste products, and mechanical stimulation of cell lines and primary cells (Legendre et al., 2013). Mimetas OrganoPlate^®^ 2-lane 96 was utilized with one in-gel culture channel and one passive perfusion channel (Lanz et al., 2017). The plate allows for the testing of multiple drugs with one patient or multiple patients with a few drugs. To better understand in which experimental conditions the organoids reached the “optimum growth rate”, different inclination degrees (7°, 15°, and 20°) and intervals of rocking including 30″, 1′, 8′, 40′ minutes, and continuous rocking were tested. As highlighted by the dotted lines in Figures 3A–C, continuous and short intervals of rocking (30″ and 1′) produced a very fast flow inside the microchannels, which probably generated high friction that destroyed the ECM. With these culture conditions, it is not possible to grow organoids as shown by the small viability values. An optimal condition for ECM and organoids was set up with an interval rocking time of 8′ and 40′ minutes (Figures 3D, E) and a maximum inclination of 7°, 15°, and 20°. In particular, the highest growth rate was reached with a tilting angle of 15° and an interval of rocking every 8 min. It is important to underline that the organoids, cultured in the above-mentioned conditions, grow better compared to a no-rocking condition (Figure 3F).

**FIGURE 4:**
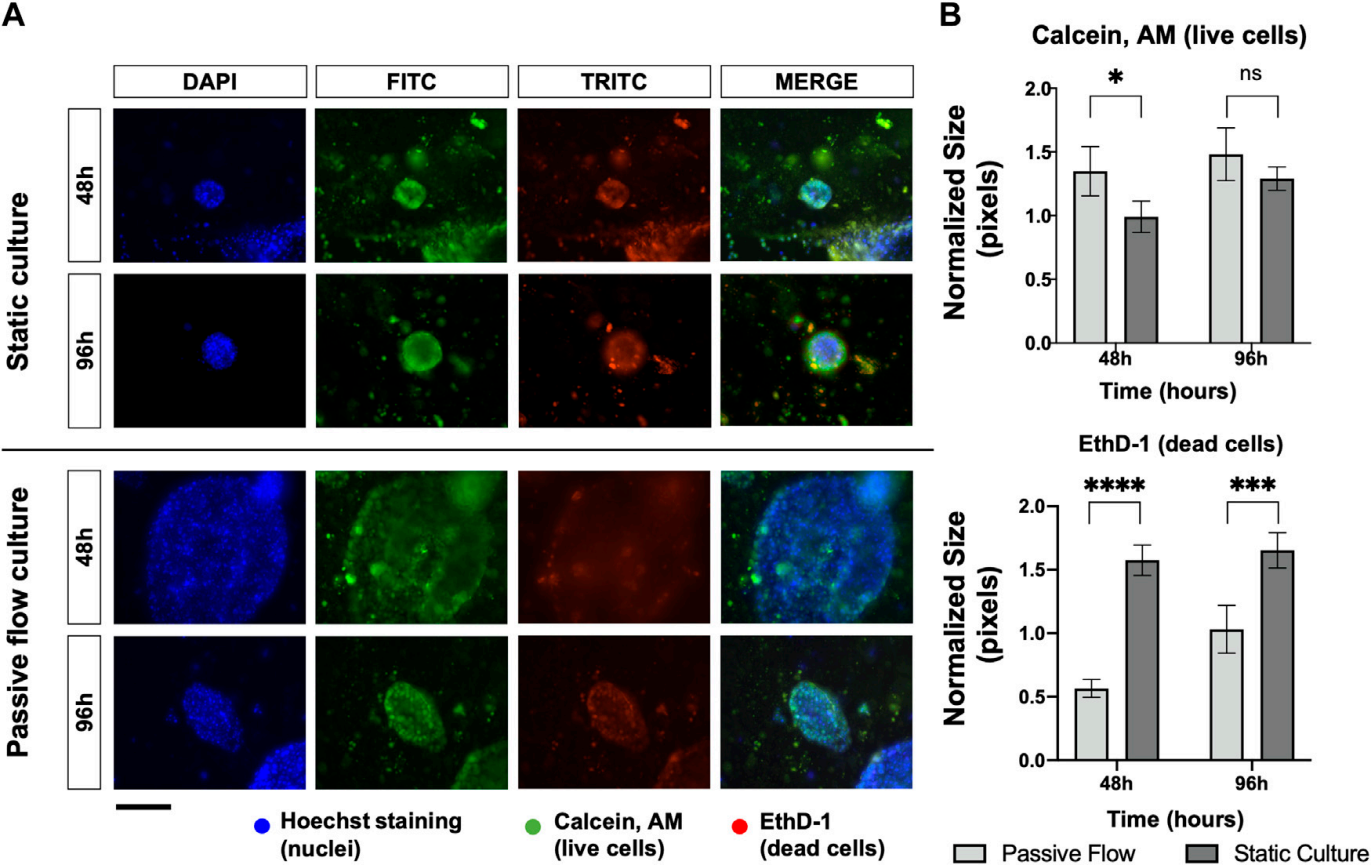
Proliferation and death rate in static and dynamic cultures: **(A)** Fluorescence microscopy images of organoids labeled with Hoescht 33,342, Calcein, AM, and EthD-1. The nuclei of the cells are shown in blue, the live cells are shown in green, and the dead cells are shown in red. A comparison at 48 and 96 h between static cultures and passive flow cultures is shown. Scalebar 250 μm; **(B)** Quantification of fluorescence signal: the number of red and green pixels was normalized by the number of blue pixels and mean ± SD is reported. The *p*-value was calculated using a two-tailed Student’s *t*-test. * *p* ≤ 0.05, ** *p* ≤ 0.01, *** *p* ≤ 0.001, and **** *p* ≤ 0.0001.

Cellular viability is mainly influenced by the rate of proliferation or the rate of cell death. In order to assess this point, organoids were stained with Calcein AM (live cells in green) and EthD-1 (dead cells in red) and observed under fluorescence microscopy. As demonstrated in Figure 4A, the microscopic analysis showed that passive flow cultures show fewer red cells and more green cells compared to static cultures. The quantification of the fluorescent signals (number of red or green pixels normalized on the number of blue pixels—Total number of cells) confirms that microfluidic technology could better reproduce the physiological conditions (Figures 4A, B).

The consistency, repeatability, and variability of the technology were evaluated in six HGOC patients. Patient 1 is an ascites derived from HGSOC. Patients 2, 3, and 5 are HGSOC chemonaive tumors, and patient 4 is a primary tumor of a high-grade endometroid ovarian cancer. All the patients were sensitive to platinum-based chemotherapy.

**FIGURE 5:**
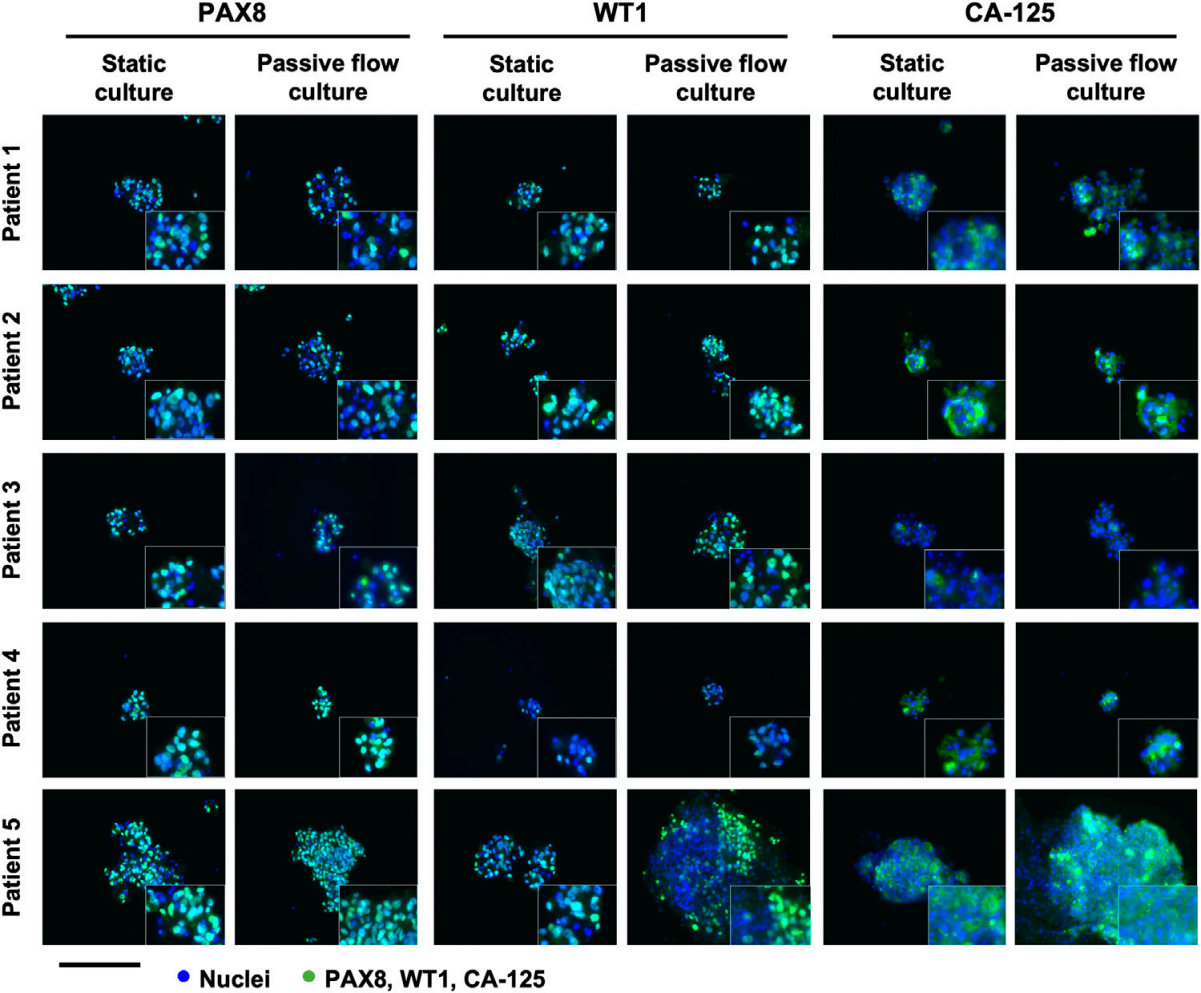
Characterization of the organoids in static and passive flow conditions: Immunofluorescent images of organoids show typical HGSOC markers: cell nuclei are in blue and cells labeled with PAX8, WT1, and CA-125 are in green. The same markers are detected in static and passive flow cultures. Inserts are cells zoomed 4×. Scalebar is 250 μm.

The established organoids captured the histological characteristics of the primary tumours. The immunohistochemistry (IHC) can be accessed as follows: Patients 1 and 4 (Adeel et al., 2021), patients 2 and 3 (paper under evaluation), and patient 5 (Supplementary Figure S3). Hematoxylin and eosin together with PAX8, WT-1, and CA-125 HGOC markers were evaluated by the pathologist. All the tissues were positive for the markers and resembled HGOC. The organoids were also characterized by immunofluorescence after being seeded in the microfluidic chip (Figure 5). PAX8, WT-1, and CA-125 HGOC markers were evaluated, and no differences were observed between static and passive flow culture conditions, indicating that organoids grown in the Mimetas 2-lane Organoplate are still similar to the parental tumors.

**FIGURE 6:**
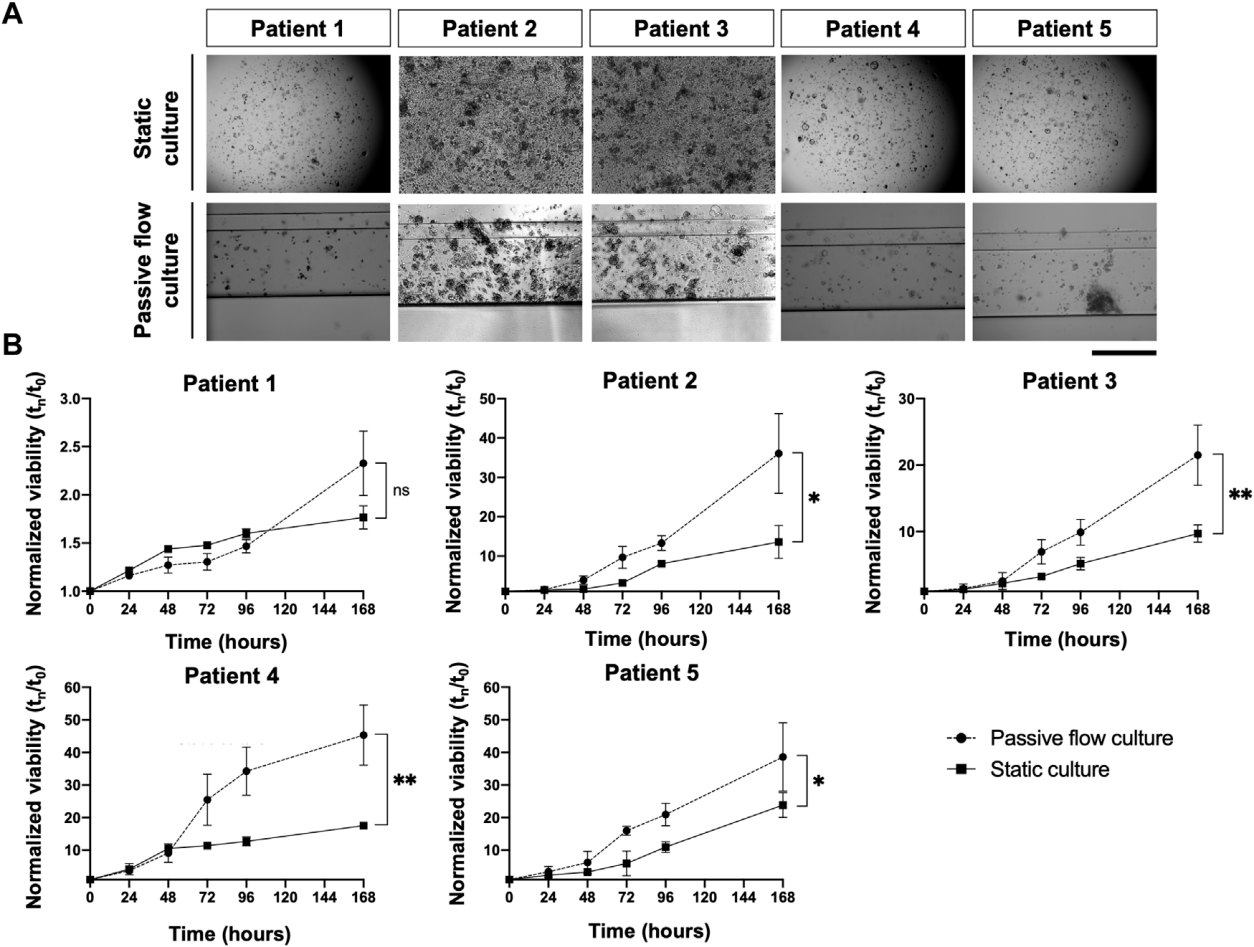
In the dynamic platform a higher growth rate is observed. **(A)** Representative bright-field images of organoids in static and passive flow cultures are shown. Scalebar 500 μm; **(B)**The growth curve of organoids derived from patients in static and passive flow conditions. Cell viability was measured using the PrestoBlue HS Assay; mean ± SD is reported, and the *p*-value was calculated using two-way ANOVA. * *p* ≤ 0.05, ** *p* ≤ 0.01, *** *p* ≤ 0.001, and **** *p* ≤ 0.0001.

The viability of the organoids seeded in the Mimetas 2-lane Organoplate was acquired for up to 7 days by measuring the power of living cells to convert resazurin to fluorescent resorufin using the PrestoBlue HS Assay. Patient 1’s growth was fast in static conditions in the first days of the culture, but after 5 days, the growth rate increased in the passive flow, and at 7 days there were no significative differences despite there being a better growth trend in passive flow conditions, even if it is not statistically significant. After 7 days, patients 2 to 5 showed a better growth rate in passive flow conditions than in static conditions (Figures 6A, B), confirming the preliminary evidence that was obtained during the setup of the culture (see Figure 3).

### Drug screening in the passive microfluidic platform

The effects of the drugs were evaluated with three different methods: the ATP and Resazurin-based method and the propidium iodide-Hoescht cell staining. In particular, the ATP method requires lysing the cellular matrix and cells of the organoids, but this did not always happen correctly due to the narrow capillaries that make up the chip structure, often generating an inadequate output. On the contrary, the Resazurin-based method is not affected by these problems because it is used directly on the culture medium. But like the method mentioned above, it requires great precision in always plating the same number of cells in order to obtain a precise (low SD) measurement of the viability. However, this is not feasible since it is not always possible to count the exact number of cells that make up a cluster of organoids, and it can happen that larger clusters can remain partially stuck in the thin channels of the Mimetas 2-lane Organoplate, making it difficult to dispense the same volume and number of organoids in the chip. In particular, the last assay was developed to overcome these problems, allowing the selection of only organoids, avoiding single cells and contaminants, and the viability is not influenced by the metabolism of the cells but only by the number of live and dead cells. It is possible to make a dose-response curve and obtain IC_50_ values even from a highly inhomogeneous sample (Supplementary Figures S1, S2). In fact, a variable number and amount (between 2 and 5 µm) of organoids were sown and treated in a serial dilution of Paclitaxel. The propidium iodide-Hoescht cell-staining method allowed us to obtain a value of IC_50_ with a much smaller standard deviation than the conventional methods (ATP-based and Resazurin-based).

**FIGURE 7:**
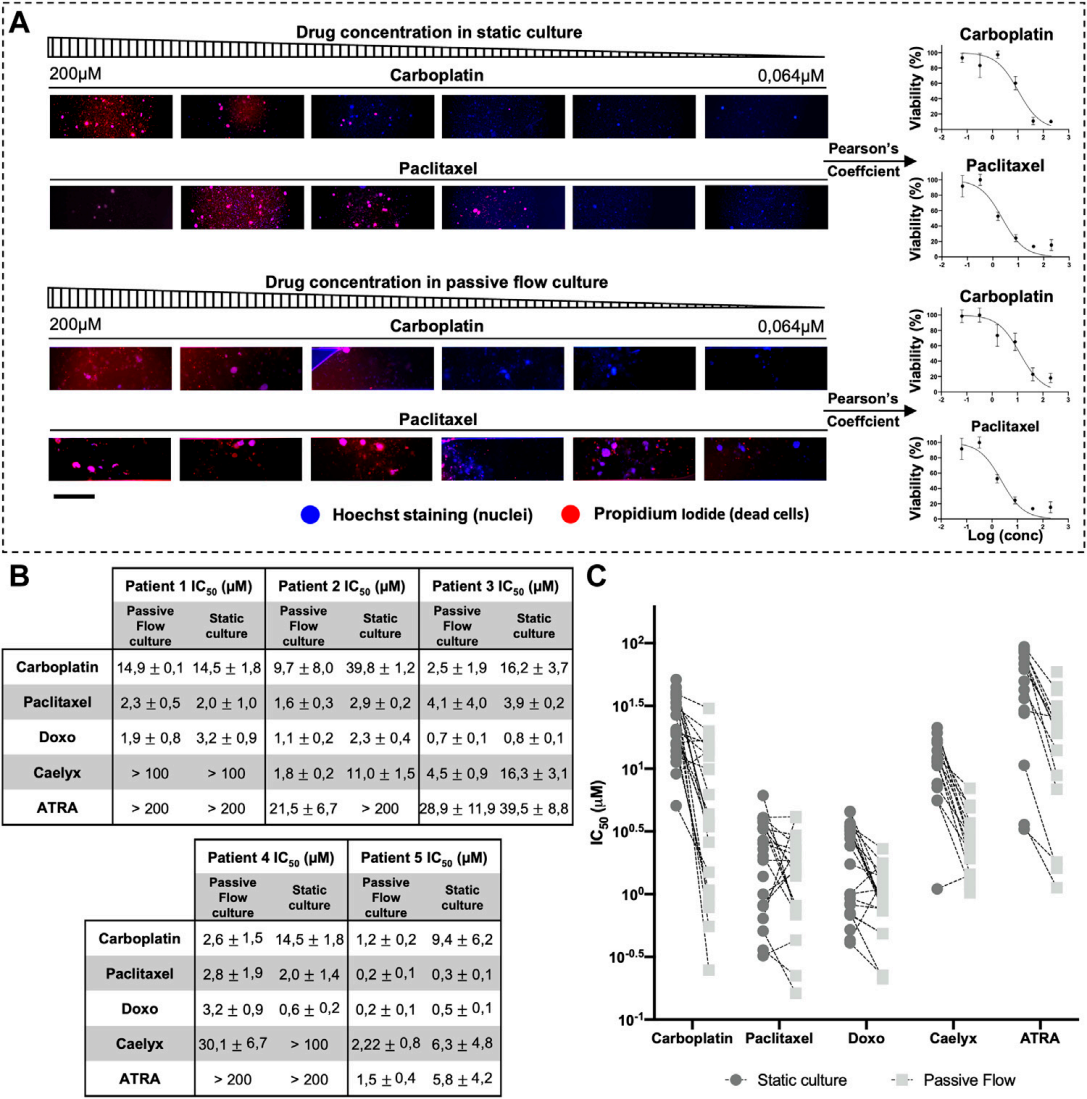
Half-maximal inhibitory concentration (IC_50_). **(A)** An example scheme of the extrapolation of a dose-response curve from Propidium iodide and hoescht-stained organoids. Organoids were treated with serial dilution of carboplatin and paclitaxel. Pearson’s coefficient was calculated and used to generate the dose-response curve. **(B)** IC_50_ Values (μM) of Carboplatin, Paclitaxel, Doxorubicin, Caelyx, and ATRA. Data were obtained from four replicates. The numbers represent the mean and standard deviation. **(C)** Graphical representation of all IC_50_ values ​​of all organoids divided by drugs. It is possible to observe how the values ​​of IC_50_ are generally smaller in the dynamic culture. Scalebar is 500 μm.

In Figure 7A, an example of PI/DAPI staining with two chemotherapeutic drugs (carboplatin and paclitaxel) is shown. In particular, for each drug concentration and each of the four replicates, TRITC-DAPI colocalization was calculated using Pearson’s coefficient. When Pearson’s Coefficient tends toward one, there is a perfect colocalization between blue (nuclei) and red (dead cell) and it means that there is 0% viability. When Pearson’s coefficient tends toward zero, it means that a lower number of red dots (dead cells) colocalizes with blue dots (nuclei), and it means that there is 100% viability. When Pearson’s coefficient tends toward minus one, it means that there is no colocalization between the red and blue dots, meaning that the paired images are not right and they get reported, which makes them particularly suitable for use in an automated system.

As shown in Figure 7B, each patient responded differently to the drugs, but there was the same consistency between the data obtained in the Mimetas 2-lane Organoplate and those obtained in the static culture. It is interesting to note that the IC_50_ values are generally smaller in the passive flow culture compared with the static conditions; a graphical representation of this observation is shown in Figure 7C (Jang et al., 2015). Of interest is the analysis of the liposomal formulation of doxorubicin (Caelyx). As a nanodrug, it improves *in vivo* pharmacokinetics and biodistribution values in the efficacy/toxicity ratio (Gabizon et al., 2003). *In vitro*, Doxil could permeate the cellular membrane slowly and then free doxorubicin, resulting in less activity, and the IC_50_ values are higher (Toffoli et al., 2015). Even the ECM represents a barrier for nanodrugs that negatively contribute to their local distribution. In this study, it was demonstrated that the passive flow could improve the activity of Doxil, and on average, the IC_50_ values are 20 times lower than in the static culture. Similarly, we tested the ATRA, which was recently discovered as a potent inhibitor of the prolyl isomerase Pin1 (Wei et al., 2015) and a target of interest for our laboratory in the therapy of ovarian cancer (Russo Spena et al., 2018). As observed for chemotherapeutic drugs, the activity of this targeted molecule improves under passive flow culture conditions.

### Drug penetration in the microfluidic chip

**FIGURE 8:**
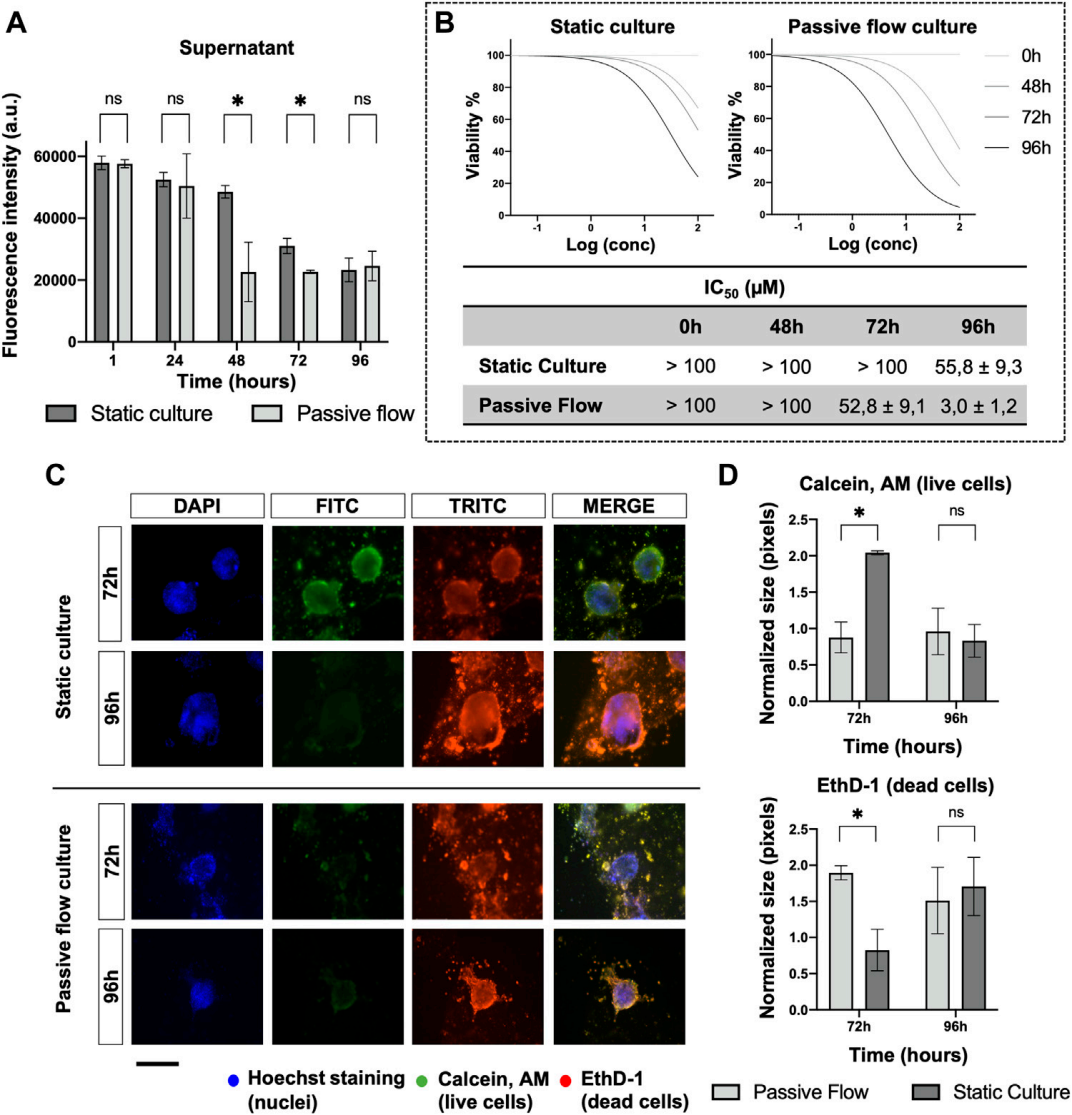
Drug penetration and organoids viability. **(A)** The amount of FITC labeled Paclitaxel is measured (in static and dynamic conditions) over time in the supernatant after 1, 24, 48, 72, and 96 h from the treatment. Mean ± SD is reported, and the *p*-value was calculated using a two-tailed Student’s *t*-test. * *p* ≤ 0.05, ** *p* ≤ 0.01, *** *p* ≤ 0.001, and **** *p* ≤ 0.0001. **(B)** Dose-response curves of organoids treated with Carboplatin. The viability has been calculated at 0, 48, 72, and 96 h from the drug treatment with the PrestoBlue HS Assay. The IC_50_ Values (μM) of Carboplatin at different time points were obtained from four replicates. The numbers represent the mean and standard deviation. **(C)** Fluorescence microscopy images of organoids labeled with Hoescht 33,342, Calcein, AM, and EthD-1. The nuclei of the cells are shown in blue, the live cells are shown in green, dead cells are shown in red. A comparison at 72 and 96 h between static cultures and passive flow cultures is shown. Scalebar 250 μm; **(D)** Quantification of fluorescence signal: the number of red and green pixels was normalized by the number of blue pixels and mean ± SD is reported. The *p*-value was calculated using a two-tailed Student’s *t*-test. * *p* ≤ 0.05, ** *p* ≤ 0.01, *** *p* ≤ 0.001, and **** *p* ≤ 0.0001.

To investigate the mechanism of drug distribution in the passive flow microfluidic, the FITC-paclitaxel was utilized, and the fluorescence was measured in the supernatant (Figure 8A) over time.

In the first 24 h, no difference was detected between the static and passive flows. But after 48 h and up to 72 h, the paclitaxel penetrated better in the ECM during passive flow conditions (Huang et al., 2012). At 96 h, again, there was no difference probably due to a saturation effect. The variation in the amount of paclitaxel that remained in the supernatant (and, consequently, the amount that penetrated the ECM) explains the data obtained in Figure 8B. To demonstrate that this effect is important for the efficacy of the drug, the *IC*_*50*_ values were calculated at different time points. In the static condition, the *IC*_*50*_ value was calculable only at 96 h. However, in the passive flow condition, the *IC*_*50*_ value was appreciable after 48 h and decreased at 72 and 96 h; the viability was calculated with the PrestoBlue HS Assay.

To directly visualize the effect of paclitaxel on organoids, Calcein AM (green, live cells) and EthD-1 (read, dead cells) were utilized for staining. As shown in Figure 8C, at 72 h, there was green staining only in static conditions. The red staining became prominent at 96 h. In passive flow conditions, green staining was not detectable but only dead cells. The quantification of green and red pixels was done as explained above and the results are reported in Figure 8D.

**FIGURE 9:**
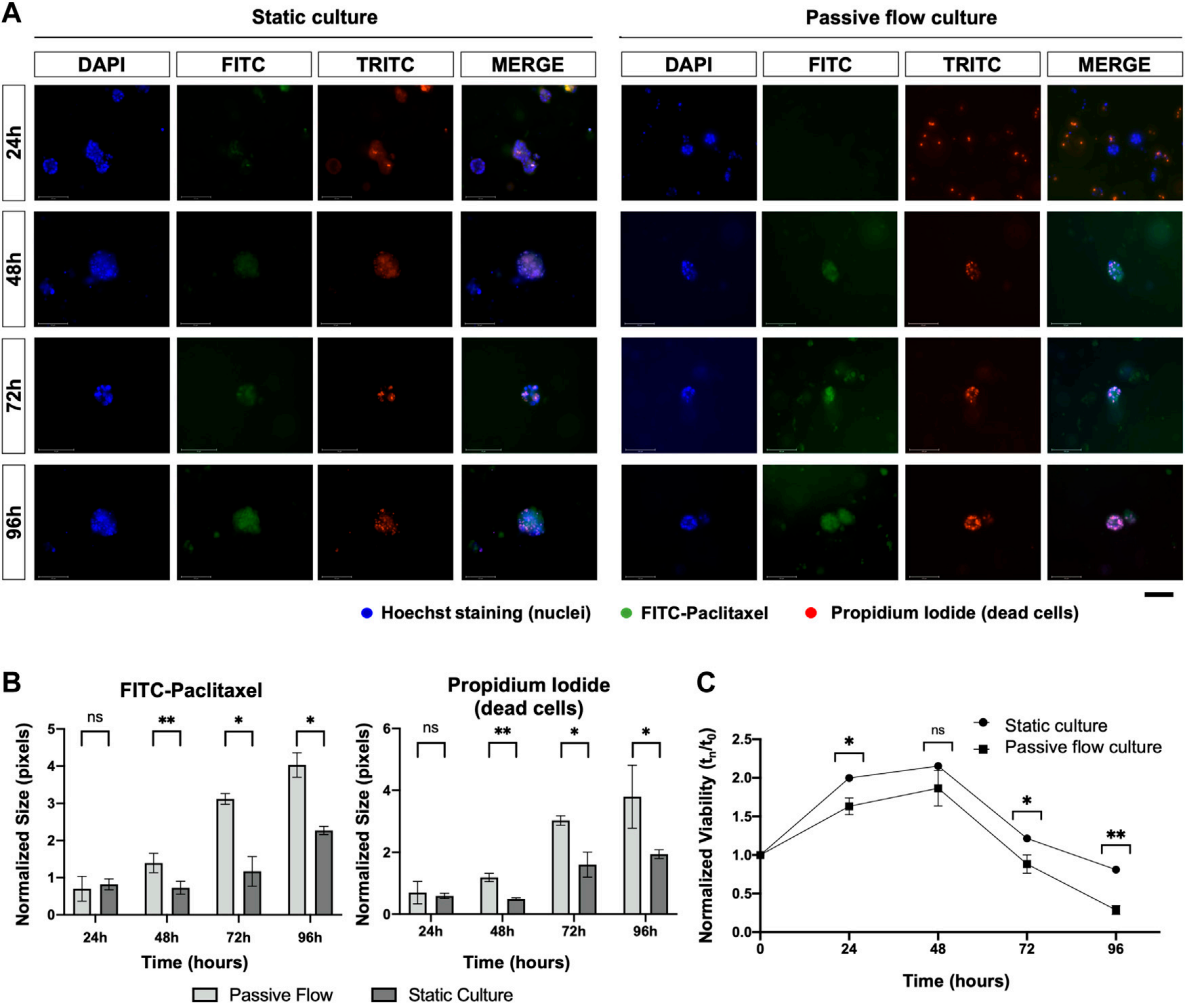
**(A)** Fluorescence microscopy images of organoids treated with FITC labeled with Paclitaxel (green), labeled with Hoescht 23,334 (nuclei, blue), and labeled with EthD-1 (dead cells, red). A comparison at 24, 48, 72, and 96 h between static cultures and passive flow cultures is shown. Scalebar 125 μm; **(B)** Quantification of fluorescence signal: the number of red and green pixels was normalized by the number of blue pixels and mean ± SD is reported. The *p*-value was calculated using a two-tailed Student’s t-test. * *p* ≤ 0.05, ** *p* ≤ 0.01, *** *p* ≤ 0.001, and **** *p* ≤ 0.0001; **(C)** The viability of the organoids treated with the FITC labeled with Paclitaxel was measured over time using the PrestoBlue HS Assay; mean ± SD is reported, and the *p*-value was calculated using a two-tailed Student’s *t*-test. * *p* ≤ 0.05, ** *p* ≤ 0.01, *** *p* ≤ 0.001, and **** *p* ≤ 0.0001.

Lastly, as shown in Figure 9, FITC-paclitaxel was utilized to treat organoids. As shown in Figure 9A, there was a greater entry of paclitaxel over time and a consequent greater cell death in dynamic conditions compared to static conditions. The amount of FITC-Paclitaxel (green) within the organoids and dead cells (red) was quantified as pixels and normalized on the numbers of the blue pixels (nuclei). Figure 9B shows that there is a correlation between a greater entry of paclitaxel into the organoids (green signal) and an increase in mortality (red signal), which is also confirmed by the viability of the organoids (Figure 9C) that decreases more over time in the dynamic cultures.

## Conclusion

It was possible to successfully cultivate organoids derived from patients with HGSOC in the Mimetas 2-lane OrganoPlate^®^. With this microfluidic platform, the organoids showed a higher proliferation rate and a lower death rate compared to static cultures. This can be attributed to the flow helping to carry nutrients and oxygen and removing toxic substances. In addition to this, it has been noted that the flow allows greater penetration of the drug into the dynamic culture, inevitably affecting cell vitality and consequently lowering the *IC*_*50*_ values compared to a static culture. This makes this technology a valid tool for drug screening in personalized medicine due to its simplicity, speed of use, and compactness. It is in fact possible to make up to 16 drugs for a patient in a single plate. Furthermore, methods based on fluorescence have been developed, which are used to work with live cells, making them available for any further analyses.

In a situation where the tumor progresses quickly and, therefore, time is essential, having a compact platform that provides a result in a short time and requires the use of a small sample is of high importance as a tool for personalized medicine.

## Data Availability

The original contributions presented in the study are included in the article/Supplementary Material, further inquiries can be directed to the corresponding author.
