# Supplementary material for: 3D dynamic cultures of HGSOC organoids to model innovative and standard therapies
Source: Front Bioeng Biotechnol. 2023 Apr 18;11:1135374. doi: 10.3389/fbioe.2023.1135374 (PMC10151532; doi:10.3389/fbioe.2023.1135374)
Supplement: Supplementary file 1 [file DataSheet1.PDF]

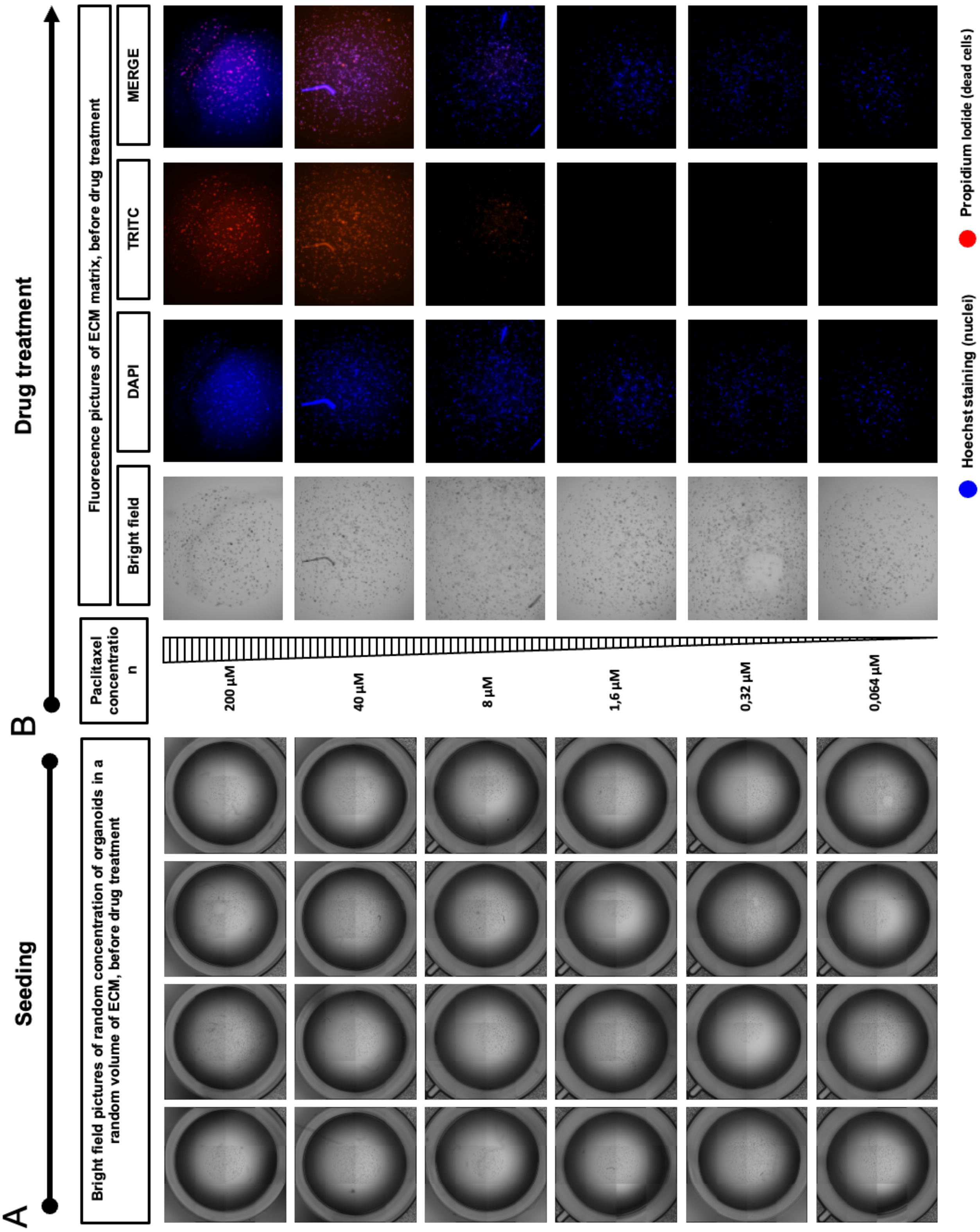

Supplementary Figure 1: *(A) Variable number and amount (between 2 and 5 µm) of organoids were sown in a 96-well plate. (B) The organoids were treated in a serial dilution of Paclitaxel. Organoids were stained with Propidium iodide-Hoescht (in blue there are nuclei, in red dead cells).*

A

Viability with PrestoBlue				
**200 µM**	6912	4698	5826	7866
**40 µM**	13985	3810	7151	5136
**8 µM**	98374	48529	96184	76314
**1,6 µM**	109393	92790	51660	50668
**0,32 µM**	96992	100707	64610	56855
**0,064 µM**	60200	50360	70280	85372

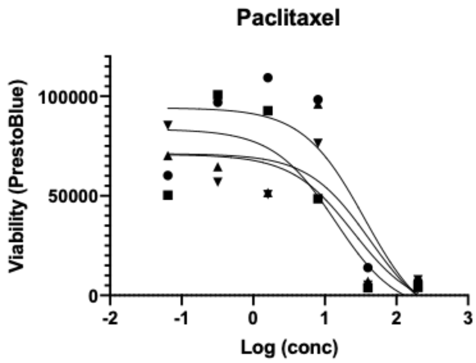

IC50 = (29,4 ± 12,7) µM

B

Pearson's Coefficient				
**200 µM**	0,914	0,902	0,919	0,903
**40 µM**	0,896	0,915	0,864	0,926
**8 µM**	0,403	0,401	0,416	0,415
**1,6 µM**	0,361	0,375	0,382	0,389
**0,32 µM**	0,327	0,299	0,302	0,322
**0,064 µM**	0,367	0,345	0,355	0,367

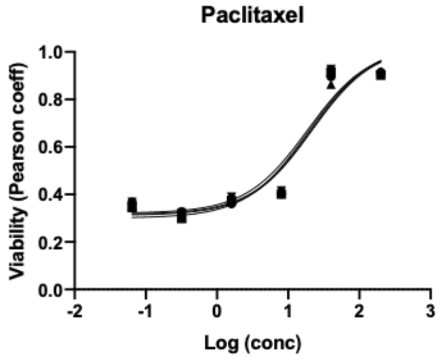

IC50 = (20,2 ± 1,5) µM

Y= 1-Pearson's Coefficient				
**200 µM**	0,086	0,098	0,081	0,097
**40 µM**	0,104	0,085	0,136	0,074
**8 µM**	0,597	0,599	0,584	0,585
**1,6 µM**	0,639	0,625	0,618	0,611
**0,32 µM**	0,673	0,701	0,698	0,678
**0,064 µM**	0,633	0,655	0,645	0,633

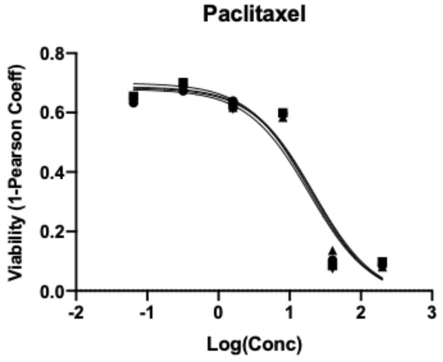

IC50 = (20,2 ± 1,5) µM

Supplementary Figure 2*: (A) Viability was evaluated with PrestoBlue, values were used to establish logistical dose-response curves and calculate the* IC_{50} *with GraphPad Prism.*

(B) The colocalization of blue areas (nuclei of organoids) and red areas (dead cells of organoids) was evaluated with Pearson's correlation coefficient using ImageJ software (see supplementary 1). Pearson's correlation coefficient was used to establish the logistical dose-response curves and calculate the IC_{50} *with GraphPad Prism. Standard deviation is ten time smaller when IC50 is calculated with the fluorescence-based method.*

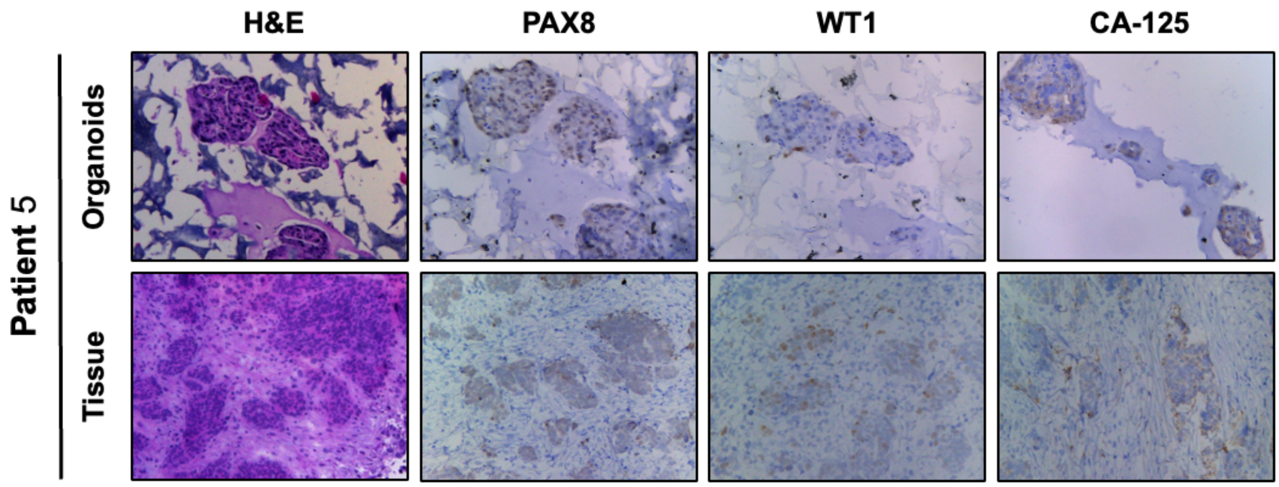

Supplementary Figure 3: *Hematoxylin & eosin staining and immunohistochemistry of HGSOC human patient (Pat 5). CA 125 (cancer antigen 125), WT1 (Wilms' Tumor 1) and PAX8 (P aired box gene 8) are markers of HGSOC*
